# Polymorphisms and haplotypes in the promoter of the TNF-α gene are associated with disease severity of severe fever with thrombocytopenia syndrome in Chinese Han population

**DOI:** 10.1371/journal.pntd.0006547

**Published:** 2018-06-25

**Authors:** Bo Xing, Xiao-Kun Li, Shao-Fei Zhang, Qing-Bin Lu, Juan Du, Pan-He Zhang, Zhen-Dong Yang, Ning Cui, Chen-Tao Guo, Wu-Chun Cao, Xiao-Ai Zhang, Wei Liu

**Affiliations:** 1 State Key Laboratory of Pathogen and Biosecurity, Beijing Institute of Microbiology and Epidemiology, Beijing, P. R, China; 2 School of Public Health, Peking University, Beijing, P. R, China; 3 The 154 Hospital, People’s Liberation Army, Xinyang, P. R, China; CDC, UNITED STATES

## Abstract

Severe fever with thrombocytopenia syndrome (SFTS) is an emerging infectious disease that is caused by a novel bunyavirus, SFTSV. We assessed whether the single nucleotide polymorphisms (SNPs) in the tumor necrosis factor-alpha (*TNF-α*) were associated with risk to severity of SFTS. Five *TNF-α* SNPs (SNP1: T-1031C; SNP2: C-863A; SNP3: C-857T; SNP4: G-308A; SNP5: G-238A) were genotyped in 987 hospitalized SFTS patients and 633 asymptomatic/mild SFTSV-infected subjects of Chinese Han origin. Multivariate logistic regression analysis was used to calculate adjusted odds ratios (ORs) and 95% confidence intervals (95% CIs). The hospitalized SFTS patients had significantly lower frequency of G-238A *A* allele than those with mild/asymptomatic infection (*P* = 0.006). Furthermore, T-1031C *C* allele (*P* < 0.001) and G-238A *A* allele (*P* < 0.001) were significantly associated with decreased risk of death. Multiple haplotypes were significantly associated with decreased risk of SFTS hospital admission (SNP1-2, CC; SNP1-3, CCC; SNP1-4, CCCG; SNP1-5, CCCGA; SNP2-4, CCGA; SNP3-5, CGA; SNP4-5, GA) and death (SNP1-2, CA; SNP1-3, CAG; SNP1-4, CACG; SNP1-5, CACGG; SNP2-3, AC; SNP2-4, ACG; SNP2-5, ACGG) after correction for multiple comparisons. By using the ELISA assay, we observed that TNF-α concentration of hospitalized patients was significantly increased in acute phase than in convalescent phase (*P* < 0.001). Elevated TNF-α concentration was also revealed from fatal patients (*P* < 0.001). The -238A allele was associated with decreased serum TNF-α levels in SFTS patients in acute phase (*P* = 0.01). Our findings suggest that polymorphisms in *TNF-α* gene may play a role in mediating the risk to disease severity of SFTS in Chinese Han population.

## Author summary

Severe fever with thrombocytopenia syndrome (SFTS) is an emerging infectious disease that is caused by a novel bunyavirus. The current study disclosed the single nucleotide polymorphisms (SNPs) in the tumor necrosis factor-alpha (*TNF-α*) were associated with risk to disease severity of SFTS. These findings suggest that polymorphisms in *TNF-α* gene may play a role in mediating the risk to disease severity of SFTS in Chinese Han population. The study will be of interest to either the clinicians devoted to the prevention and therapy of SFTS or the geneticists devoted to studying the genetic susceptibility mechanisms of common diseases.

## Introduction

Severe fever with thrombocytopenia syndrome (SFTS) is an emerging infectious disease that is caused by a novel bunyavirus named SFTS virus (SFTSV) [1], a novel phlebovirus belongs to the *Phenuiviridae* family (https://talk.ictvonline.org/taxonomy). Since its discovery in 2009, over three thousands of cases have been reported from at least 23 provinces in China [2]. Recent case report in South Korea and Japan demonstrated its existence outside of China, thus indicating the imminent public health impact of this emerging infectious disease [3–5].

Host genetic variations may contribute to severity and death of SFTS. Although large amounts of individuals had been exposed to the SFTSV in endemic areas, only a small proportion developed symptomatic disease, with their clinical manifestations ranging widely from an acute self-limited febrile illness to complications of various severity and even death [6,7]. Among all the studies that reported risk factors for adverse disease outcome, older age has been consistently found to increase the death risk [8,9], suggesting the role of host immunity in determining the clinical disease.

Based on the current knowledge, inflammatory cytokines and chemokines, the first ramification of activation of the innate immune cells, play important roles in the pathogenesis of SFTS [10–12]. As for patients with adverse disease outcome, the altered production of these cytokines has constantly been found, which process might be genetically determined [13]. Actually, both peripheral cytokine level and their determined genetic polymorphism have been explored for their relationship with the risk of acquiring infection and related disease severity, such as hepatitis B and fungal infections [14,15]. Among the cytokines, TNF-α is a major regulator of the inflammatory response that acts locally to trigger a cascade of other pro-inflammatory and chemotactic cytokines and adhesion factors. TNF-α has been putatively implicated in the pathogenesis of a variety of diseases including infectious disease, autoimmune disorders, neoplasia, and malignant diseases [16]. The increased levels of TNF-α was observed in SFTS patients than in healthy individuals, and to an even higher level in fatal patients [17–19]. A number of single nucleotide polymorphisms (SNPs), which are thought to affect the TNF-α production, have been found to alter individual susceptibility to a wide spectrum of infectious disease. Among all the genetic polymorphisms that determined the TNF-α expression in human serum, those located in the promoter region have been most frequently implicated in the regulation of TNF-α expression [20–22]. On the basis of the functional role of *TNF-α* in the pathogenesis of SFTS, we are impelled to explore the possible role of the *TNF-α* promoter polymorphisms in determining the disease severity of SFTS in Chinese Han population.

## Results

### Population characteristics

A total of 987 virologically confirmed SFTS patients who needed hospitalization and 633 asymptomatic/mild SFTSV-infected subjects of Chinese Han origin were recruited for the study. Fatal outcome developed in 106 hospitalized SFTS patients. By checking the medical records and by interviewing the participants’ guardians, we determined that all cases and controls were genetically unrelated Han Chinese. The selected characteristics of subjects are shown in Table 1. Compared with the asymptomatic/mild SFTSV-infected subjects, SFTS hospitalized patients were significantly older (*P* < 0.001), more often to be female (*P* < 0.001) and with more presence of underlying medical conditions (*P* = 0.009). Compared with non-fatal patients, significantly older age (*P* < 0.001), more male gender (*P* = 0.046) and over-presence of underlying medical conditions (*P* = 0.001) were found in fatal patients (Table 1).

**Table 1:** Selected characteristic of hospitalized patients with severe fever with thrombocytopenia syndrome and asymptomatic/mild SFTSV-infected subjects.

Variables	Hospitalized patients			Asymptomatic/mild SFTSV-infected subjects (n = 633)	*P* value^a^	
	Subtotal (n = 987)	Non-fatal (n = 881)	Fatal (n = 106)		Hospitalized patient vs. asymptomatic/mild SFTSV-infected subjects	Non-fatal vs. fatal patients
Age, year						
Mean (SD)	60.7 (12.1)	59.9 (12.0)	67.4 (10.5)	49.5 (16.0)	< 0.001	< 0.001
≤ 60, n. (%)	457 (46.3)	437 (49.6)	20 (18.9)	468 (73.9)	< 0.001	< 0.001
Male, n (%)	419 (42.5)	364 (41.3)	55 (51.9)	346 (54.7)	< 0.001	0.046
Underlying medical conditions^b^, n (%)	272 (27.6)	228 (25.9)	44 (41.5)	138 (21.8)	0.009	0.001

Abbreviations: SD, standard deviation.

^a^*χ*^2^ test for categorical variables and the Mann Whitney U test for continuous variables.

^b^The underlying medical conditions were defined as patients presenting with one of the following: hypertension, diabetes, cancer, active hepatitis, cerebral infarction, et al.

### Individual polymorphism and risk of hospital admission of SFTS

Sequencing of the ~1.2-kb genomic region in the *TNF-α* gene in 174 individuals revealed 8 polymorphisms (Table 2). To ensure enough statistical power, a value of 0.03 of minor allele frequency was set as the threshold value of inclusion in this study. Finally, five polymorphisms (SNP1, T-1031C; SNP2, C-863A; SNP3, C-857T; SNP4, G-308A; and SNP5, G-238A) were selected in the subsequent genotyping analysis.

**Table 2:** Positions and frequencies of polymorphisms screened from the tumor necrosis factor-alpha (*TNF-α*) gene.

No.	Polymorphisms	dbSNP ID numbers	Position^a^	Minor allele frequency	Regions
1	T-1031C	rs1799964	3064	0.194	Promoter
2	C-863A	rs1800630	3232	0.156	Promoter
3	C-857T	rs1799724	3238	0.087	Promoter
4	C-806T	rs4248158	3289	0.014	Promoter
5	A-572C	rs4248161	3523	0.024	Promoter
6	G-376A	rs1800750	3719	0.010	Promoter
7	G-308A	rs1800629	3787	0.059	Promoter
8	G-238A	rs361525	3857	0.045	Promoter

^a^The positions of the polymorphisms are relative to the initial site of transcription of the *TNF-α* gene (GenBank accession no. M16441.1).

The genotyping results for the five *TNF-α* polymorphisms were shown in Table 3. The observed genotype frequencies for the five polymorphisms conformed to Hardy-Weinberg equilibrium in two groups, respectively (all *P* > 0.05). When compared with asymptomatic/mild SFTSV-infected subjects controls, significantly decreased frequencies of SNP1 and SNP5 were observed in hospitalized SFTS patients by using multivariate logistic regression model to adjust for the effect from age, sex, and underlying medical conditions (*P* = 0.043 and 0.006 respectively) (Table 3). After multiple corrections, only G-238A was significantly associated with hospital admission of SFTS.

**Table 3:** The genotype frequencies of *TNF-α*polymorphisms in hospitalized patients with severe fever with thrombocytopenia syndrome and asymptomatic/mild SFTSV-infected subjects.

SNPs and genotypes	Hospitalized patients (n = 987)	Asymptomatic/mild SFTSV-infected subjects (n = 633)	OR (95% CI) ^a^	*P* value ^a^
SNP1 (T-1031C)				
TT	649 (66.5)	388 (62)	Reference	
TC	300 (30.7)	209 (33.4)	0.83 (0.65–1.04)	0.05
CC	27 (2.8)	29 (4.6)	0.54 (0.30–0.97)	
TC+CC	327 (33.5)	238 (38)	0.79 (0.63–0.99)	0.043
SNP2 (C-863A)				
CC	715 (73.4)	448 (71.5)	Reference	
CA	242 (24.9)	170 (27.1)	0.88 (0.69–1.13)	0.59
AA	17 (1.7)	9 (1.4)	1.12 (0.47–2.63)	
CA+AA	259 (26.6)	179 (28.5)	0.90 (0.71–1.14)	0.38
SNP3 (C-857T)				
CC	751 (77.1)	482 (76.9)	Reference	
CT	209 (21.5)	135 (21.5)	1.03 (0.79–1.33)	0.98
TT	14 (1.4)	10 (1.6)	1.01 (0.41–2.50)	
CT+TT	223 (22.9)	145 (23.1)	1.02 (0.79–1.32)	0.85
SNP4 (G-308A)				
GG	868 (88.8)	548 (86.7)	Reference	
GA	109 (11.1)	81 (12.8)	0.76 (0.54–1.06)	0.10
AA	1 (0.1)	3 (0.5)	0.20 (0.02–2.04)	
GA+AA	110 (11.2)	84 (13.3)	0.74 (0.53–1.02)	0.07
SNP5 (G-238A)				
GG	923 (94.5)	571 (90.3)	Reference	
GA	54 (5.5)	60 (9.5)	0.58 (0.38–0.88)	0.005
AA	0 (0)	1 (0.2)	NA	
GA+AA	54 (5.5)	61 (9.7)	0.56 (0.37–0.85)	0.006

NOTE: The number of genotyped samples varies because of genotyping failure for some individuals.

Abbreviations: OR, odds ratio; CI, confidence interval; NA, not applicable.

^a^The ORs and *P* values were adjusted for age, sex, and underlying medical conditions.

The associations between the G-238A polymorphism and hospital admission of SFTS were further examined with stratification by age, sex, and underlying medical conditions (S1 Table). Although the effect appeared to be more pronounced in subjects who were females, younger (≤60 years), and without underlying medical conditions, these differences could be attributed to chance (all *P* > 0.07, test for homogeneity), indicating that these potential confounding factors had no modification effect on the risk of SFTS hospital admission related to the G-238A genotypes.

### Individual polymorphism and risk of SFTS related death

By using multivariate logistic regression model to adjust for the effect from age, sex, and underlying medical conditions, significant associations with fatal outcome were observed for the T-1031C and G-238A polymorphisms (Table 4). For T-1031C polymorphism, the genotypes containing *C* allele (TC + CC genotypes) were significantly associated with decreased risk to death when compared with the TT genotype (OR = 0.43, 95% CI = 0.26–0.71; *P* < 0.001) (Table 4). For G-238A polymorphism, when compared with the -238GG genotype, the genotypes containing *A* allele (GA + AA genotypes) were significantly associated with decreased risk to SFTS related death (*P* < 0.001). The significant associations remained after correction for multiple comparisons. No association between risk of SFTS related death and other investigated polymorphisms were found after multiple testing. In the stratification analyses, sex, age, and underlying medical condition had no modification effect on the risk of SFTS related death related to the -1031 TC + CC genotypes and -238 GA + AA genotypes respectively (S2 Table).

**Table 4:** The genotype frequencies of *TNF-α*polymorphisms in non-fatal and fatal patients with severe fever with thrombocytopenia syndrome.

SNPs and genotypes	Non-fatal patients (n = 881)	Fatal patients (n = 106)	OR (95 CI)^a^	*P*^a^
SNP1 (T-1031C)				
TT	566 (64.9)	83 (79.8)	Reference	
TC	279 (32)	21 (20.2)	0.47 (0.28–0.79)	< 0.001
CC	27 (3.1)	0 (0)	NA	
TC+CC	306 (35.1)	21 (20.2)	0.43 (0.26–0.71)	< 0.001
SNP2 (C-863A)				
CC	629 (72.3)	86 (82.7)	Reference	
CA	224 (25.8)	18 (17.3)	0.59 (0.34–1.01)	0.019
AA	17 (2)	0 (0)	NA	
CA+AA	241 (27.7)	18 (17.3)	0.55 (0.32–0.94)	0.021
SNP3 (C-857T)				
CC	668 (76.8)	83 (79.8)	Reference	
CT	189 (21.7)	20 (19.2)	0.87 (0.52–1.48)	0.83
TT	13 (1.5)	1 (1)	0.69 (0.09–5.46)	
CT+TT	668 (76.8)	83 (79.8)	0.86 (0.51–1.44)	0.57
SNP4 (G-308A)				
GG	772 (88.4)	96 (91.4)	Reference	
GA	100 (11.4)	9 (8.6)	0.65 (0.31–1.36)	0.41
AA	1 (0.1)	0 (0)	NA	
GA+AA	772 (88.4)	96 (91.4)	0.64 (0.31–1.34)	0.22
SNP5 (G-238A)				
GG	819 (93.8)	104 (100)	Reference	
GA	54 (6.2)	0 (0)	NA	< 0.001
AA	0 (0)	0 (0)	NA	
GA+AA	54 (6.2)	0 (0)	NA	< 0.001

The number of genotyped samples varies because of genotyping failure for some individuals.

Abbreviations: OR, odds ratio; CI, confidence interval; NA, not applicable.

^a^Comparison between non-fatal patients and fatal patients, after adjusted for age, sex, and underlying disease.

### Haplotypes and SFTS disease severity

**Fig 1:**
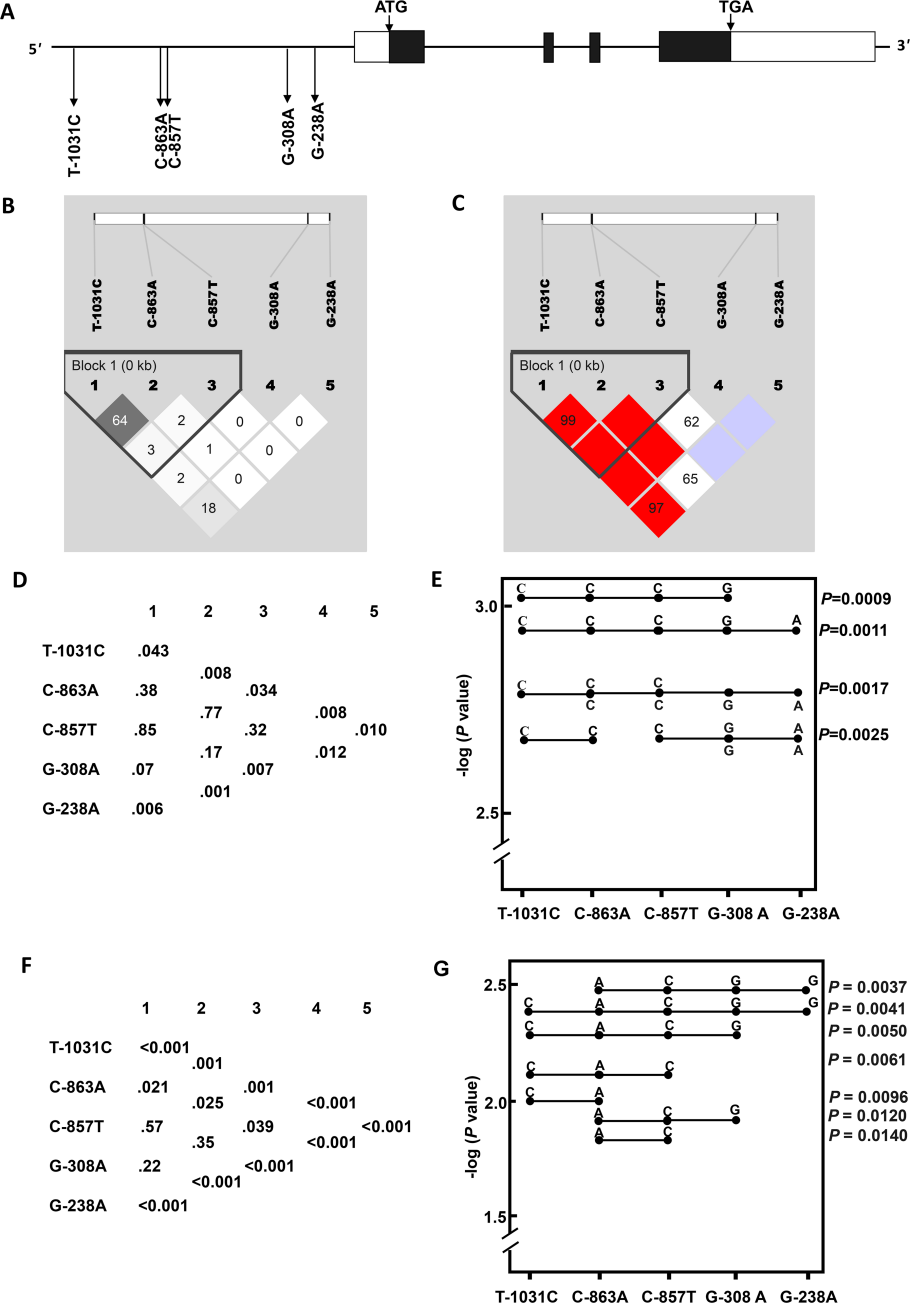
Haplotypes of *TNF-α* polymorphisms and the risk for hospital admission and death of severe fever with thrombocytopenia syndrome (SFTS). (A) Genomic structure of the *TNF-α* locus and five polymorphic sites used. Exons (boxes) and introns are not drawn to scale; open boxes represent noncoding sequences, filled boxes represent coding sequences. (B) Linkage disequilibrium (LD) map of SNPs based on the *D*´. (C) LD map of SNPs based on *r*^2^. (D) Global *P* values from single-locus and multilocus (two to five) association analysis and haplotypes with significant association between hospitalized SFTS patients and asymptomatic/mild SFTSV-infected subjects. (E) Global *P* values from single-locus and multilocus association analysis and haplotypes with significant association for hospital admission of SFTS. (F) Global *P* values from single-locus and multilocus (two to five) association analysis and haplotypes with significant association between non-fatal and fatal SFTS patients. (G) Global *P* values from single-locus and multilocus association analysis and haplotypes with significant association for death of SFTS.

The pairwise disequilibria measures (*D*´ and *r*^2^) of the five *TNF-α* polymorphisms (Fig 1A) were calculated. Fig 1B and 1C showed that two polymorphisms, T-1031C and C-863A, were in strong LD. We next performed haplotype analysis to derive haplotypes specifically correlated with disease severity of SFTS. When compared with asymptomatic/mild SFTSV-infected subjects, the frequencies of seven multi-SNP haplotype systems (Fig 1D) and multiple haplotypes (SNP1-2, CC; SNP1-3, CCC; SNP1-4, CCCG; SNP1-5, CCCGA; SNP2-4, CCGA; SNP3-5, CGA;SNP4-5, GA) (Fig 1E) were found to be significantly lower in hospitalized SFTS patients after correction for multiple comparisons.

When comparison was made between non-fatal and fatal patients, several multi-SNP haplotype systems (Fig 1F) and seven multi-SNP haplotypes (SNP1-2, CA; SNP1-3, CAG; SNP1-4, CACG; SNP1-5, CACGG; SNP2-3, AC; SNP2-4, ACG; SNP2-5, ACGG) were found to be associated with decreased susceptibility to death of SFTS, after correction for multiple comparisons (Fig 1G).

### Effects of the T-1031C and G-238A polymorphisms on TNF-α serum expression

**Fig 2:**
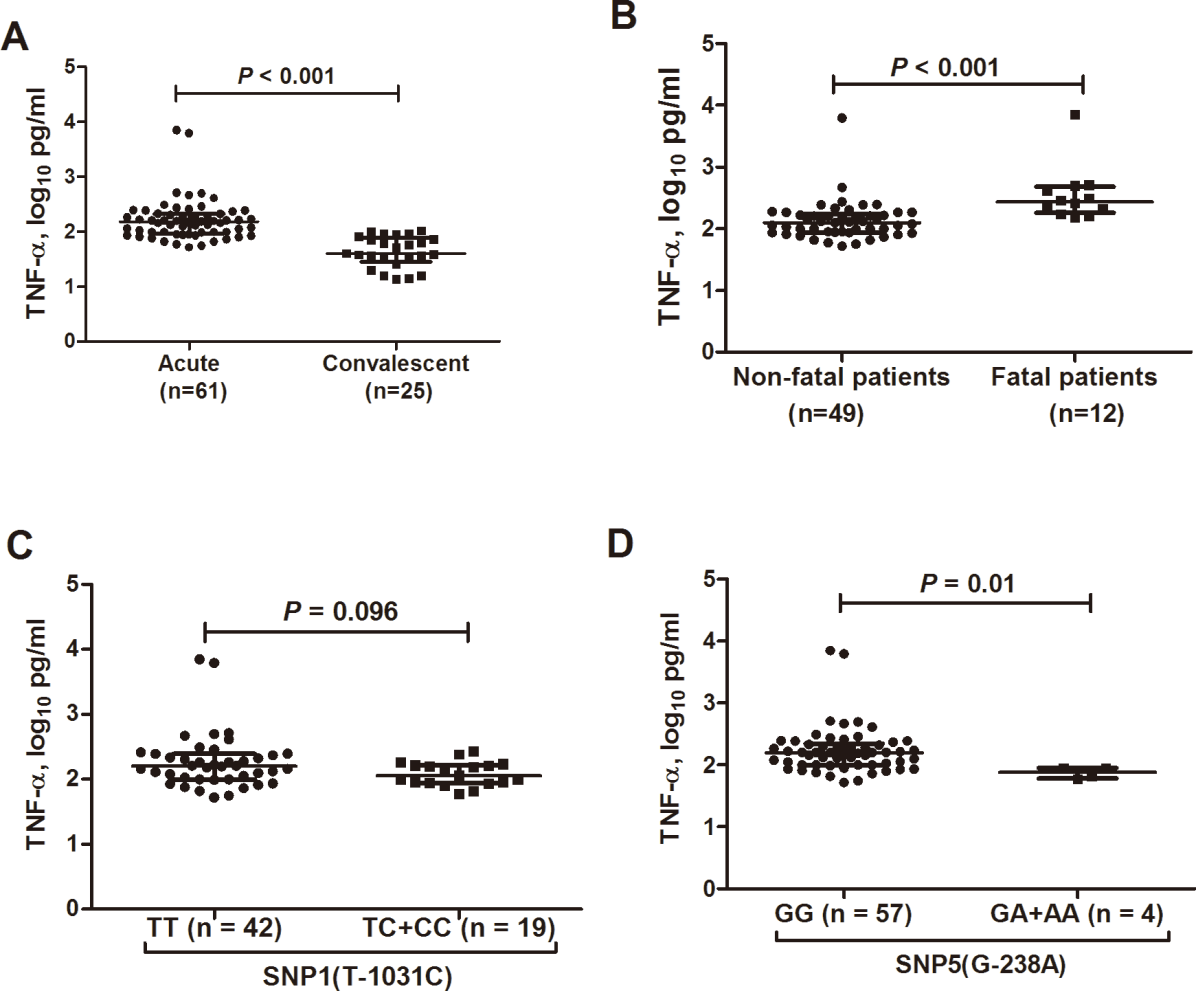
*TNF-α* polymorphisms and TNF-α expression among severe fever with thrombocytopenia syndrome patients with different outcomes. (A) Serum TNF-α levels between acute phase and convalescent phase of hospitalize SFTS patients. (B) Serum TNF-α levels between SFTS patients with fatal and nonfatal outcome. (C) Correlation of TNF-a serum expression with SNP1 genotypes in hospitalized SFTS patients in acute phase. Compared to the TT carriers, the *C* allele carriers had had a comparable TNF-a serum expression (*P* = 0.096). (D) Correlation of TNF-a serum expression with SNP5 genotypes in hospitalized SFTS patients in acute phase. Compared to the GG carriers, the *A* allele carriers had a markedly lower TNF-a serum expression (*P* = 0.01).

Altogether 61 hospitalized SFTS patients at acute phase and 25 hospitalized SFTS patients at convalescent phase were evaluated for the serum TNF-α level. The serum TNF-α levels from acute phase were significantly higher than that obtained from convalescent phase (*P* < 0.001; Fig 2A). In addition, the TNF-α levels from fatal hospitalized SFTS patients were significantly increased compared with non-fatal hospitalized SFTS patients (*P* < 0.001; Fig 2B).

The TNF-α serum levels in 61 SFTS patients were also evaluated for their association with T-1031C and G-238A genotypes. No significant difference of TNF-α serum levels was observed between T-1031C TC + CC genotypes and TT genotype carriers (*P* = 0.096; Fig 2C). However, among the 61 hospitalized SFTS patients, those carrying the -238A allele (n = 4) had significantly lower TNF-α level than the GG genotype carriers (n = 18) at acute phase (*P* = 0.01; Fig 2D).

## Discussion

In this study, we found two SNPs (T-1031C and G-238A) in the promoter of *TNF-α* gene were associated with disease severity of SFTS in Chinese Han population. Multi-SNP haplotypes derived from the *TNF-α* polymorphisms was also shown to be associated with the decreased risk to hospital admission and death of SFTS. Furthermore, consistent with the population-based association study, the decreased TNF-α serum levels from the -238A carriers were also observed. These findings suggest that *TNF-α* gene polymorphisms might contribute to the severity of SFTS by influencing TNF-α expression in Chinese Han population.

Our observed genetic associations are plausible from a biological perspective. TNF-α is a potent pro-inflammatory and immunoregulatory cytokine that plays a key role in the initiation, regulation, and perpetuation of the inflammatory response. As for SFTS, we found that the TNF-α concentration of hospitalized patients was higher in acute phase as than in convalescent phase. Elevated TNF-α concentration was also revealed from fatal patients. Our results are consistent with those of previous studies, regarding the abnormally increased expression of TNF-α in the severe and especially fatal SFTS [10–12, 17–19]. TNF-α has been suggested to act on the endothelium, inducing vasodilating substances, stimulating nitric oxide synthase, increasing capillary endothelial permeability. This process might be responsible for the occurrence of haemorrhagic manifestations, eventually resulting in DIC or MOF, and even death in SFTS [23].

The *TNF-α* T-1031C and G-238A polymorphisms are reportedly capable of altering TNF-a expression, however with controversy among various studies. In vitro studies showed that the -1031C and -238A alleles conferred increased transcriptional activation of the TNF promoter [20,24,25]. In contrast, other studies showed no associations between two polymorphisms and TNF-α expression [21,22]; one study revealed that the -238A-allelic *TNF-α* promoter was associated with a reduced transcriptional activity by luciferase assays and this allele was associated with decreasing TNF-α expression in psoriasis patients [26]. In the present study, we indeed found decreased TNF-α serum levels in hospitalized SFTS patients with the -238A allele, which is consistent with the results of population-based association study, however, no such significant association was determined between T-1031C genotypes and TNF-α levels. Further studies with large sample size are warranted to elucidate the molecular mechanism of these two important polymorphisms.

This study demonstrated how the appropriate choice of control groups might impact on the results of population based study. The genius control group might be mixed up with individuals who have not been exposed to the virus at all, thus masking the association. The current study chose asymptomatic SFTSV-infected subjects as controls. The asymptomatic SFTSV-infected controls might represent a real control, who have been similarly challenged with the SFTSV, while remained apparently healthy, or at least with very mild disease not to be recalled by the individual. Because asymptomatic SFTSV-infected subjects cannot necessarily recall all the possible flu-like symptoms that might be a mild or very mild SFTS disease within a period of 5 years, we have defined the asymptomatic SFTSV-infected subjects as asymptomatic/mild SFTSV-infected subjects.

Recently, several association studies have shown that the *TNF-α* polymorphisms were related to the susceptibility to various specific infections, including pulmonary tuberculosis, leprosy, severe sepsis in trauma patients, HBV, and HIV [27–32]. Some of the results, however, could not be replicated in subsequent studies. The lack of reproducibility may be ascribed to multiple factors, such as small sample sizes, the different ethnicities of study populations and/or different genetic background. The design and results of our study include many of the features that are considered desirable components of an ideal association study, including large sample size, small *P* values, and an association that makes biological sense.

We acknowledge the potential limits of the study. Firstly, due to the low minor allele frequency of G-238A, the number of the -238A allele carriers for the ELISA assay was very small. Consequently, the effects of the G-238A polymorphism on TNF-α serum expression should be interpreted in caution. Secondly, considering that lower level of TNF-α expression in -238A allele was associated with decreased risk to severe SFTS, G-238A cannot be used to identify individuals with high-risk of becoming severe. Thirdly, only five polymorphisms in the *TNF-α* promoter were studied. Without performing a systematic screen for variants in the whole *TNF-α* gene, we cannot exclude the possible linkage disequilibrium that existed between these two polymorphisms and other nearby causative variant. Deep resequencing of this gene may help to uncover additional associated variants and facilitate selection of potential causal variants for further functional studies.

In conclusion, our results reveal, for the first time, an association between the *TNF-α* polymorphisms and lower risk to severe of SFTS in Chinese Han population. These findings provided evidence supporting the importance of TNF-α in the pathogenesis of SFTS. If confirmed by other studies, knowledge of genetic factors contributing to the pathogenesis of SFTS as presented here would be important for the assessment of one’s susceptibility to SFTS and other infectious diseases, especially those sharing a mode of action similar to that of SFTS.

## Methods

### Study populations

The study was performed in a SFTS designated hospital (The 154 Hospital of People’s Liberation Army) in Xinyang administrative district of Henan Province between 2011 and 2014. All SFTS patients were newly diagnosed and virologically confirmed hospitalized patients. Definitive diagnosis of SFTS patients was based upon typical clinical and epidemiological findings and by detection of SFTSV genomic segments using reverse transcription polymerase chain reaction (RT-PCR) (detailed in SFTSV RNA detection). Information regarding demographic characteristics, medical history, clinical manifestation, laboratory test results were prospectively collected using a standard questionnaire.

Asymptomatic/mild SFTSV-infected subjects (no need of medical attention at a hospital) were selected from healthy subjects who underwent routine physical examination in the same hospital during the same period when the cases were recruited. Their sera samples were subjected for SFTSV specific IgG antibody test by enzyme-linked immunosorbent assay (ELISA). Only those positive for SFTSV IgG antibody while negative for SFTSV genomic segments and denied clinical manifestations resembling SFTS were included as eligible asymptomatic/mild SFTSV-infected subjects. By checking the medical records or by interviewing the participants, we determined that all asymptomatic/mild SFTSV-infected subjects were genetically unrelated Han Chinese and have not been hospitalized for febrile disease in the past five years.

For each participant, peripheral blood and sera were collected and immediately stored at -80°C until genomic DNA/RNA extraction.

### Ethics statement

The study was performed with the approval of the Ethical Committee of Beijing Institute of Microbiology and Epidemiology and conducted according to the principles expressed in the Declaration of Helsinki. All participants were adults and provided written informed consent.

### SFTSV RNA detection

Viral RNA was isolated from serum samples using QIAamp Viral RNA Mini Kit (Qiagen, Germantown, MD, USA), according to the manufacturer’s instructions. One step Primer Script RT-PCR Kit (TaKaRa) was used according to the manufacturer’s instructions for SFTSV detection according to the method described previously [33]. The reaction was performed on an ABI 7500 Real Time PCR System (Applied Biosystems, USA). The primers and TaqMan probes used for the SFTSV detection were as follows: 5′-TTCACAGCAGCATGGAGAGG-3′ (forward primer), 5′-GATGCCTTCACCAAGACTATCAATG-3′ (reverse primer), 5′-AACTTCTGTCTTGCTGGCTCCGC-3′ (probe). Nested RT-PCR and sequencing of the M- segment were performed on randomly selected positive samples to verify the real-time RT-PCR results.

### Validation of *TNF-α* variants

The primer set covering the genomic sequence of the promoter region of the *TNF-α* gene, which spans 1.2 kb (from nt 2935 to nt 4137; GenBank accession no. M16441.1), was designed on the basis of size and overlap of polymerase chain reaction (PCR) amplicons. The screening panel included DNA from 174 individuals randomly selected, without regard to disease status, from the total study population of 2270 individuals. The primers for the target regions were designed using the Web-based software Primer3 [34, 35] (S3 Table). DNA samples from the 174 individuals were amplified and purified. PCR conditions were identical to those used for the SNP discovery described previously [36]. Briefly, PCR was performed with a 25 mL reaction mixture containing 20ng DNA, 1.0mmol/L each primer, 0.2 mmol/L each dNTP, 2.0 mmol/L MgCl2, and 1.0 U Taq DNA polymerase in 1X reaction buffer (Takara Biotech, Dalian, China). The reaction for amplification was carried out in the following conditions: an initial melting step of 2 min at 95°C, followed by 35 cycles of 30 s at 94°C, 30 s at 57°C, and 30 s at 72°C and a final elongation of 7 min at 72°C. Then the PCR products were sequenced using an ABI PRISM Dye Terminator Sequencing Kit with Amplitaq DNA polymerase (ABI) and loaded onto an ABI 3730 sequencer. Polymorphism candidates were identified by the PolyPhred program and were inspected by 2 observers. Polymorphism positions and individual genotypes were confirmed by reamplifying and resequencing the polymorphism sites from the opposite strand. The primers are available on request.

### Polymorphism genotyping

The five promoter polymorphisms were selected for genotyping by use of PCR direct sequencing in the case-control population. The primers for PCR and sequencing and the reaction parameters were identical with those used for the polymorphism validation procedure mentioned above. Genotyping was done in a blind manner that the performers did not know the subjects’ case and control status. The accuracy of genotyping data for each polymorphism was validated by masking, choosing at random, and resequencing 15% of the samples from case patients and control subjects.

### ELISA assay

To compare the differential expression of TNF-α among genotypes, hospitalized SFTS patients and asymptomatic/mild SFTSV-infected subjects were randomly selected to measure the serum concentrations of TNF-α by using TNF-α ELISA assay (GenWay Biotech, USA). The assays were performed according to the instructions of the manufacturers. All measurements were performed in duplicate.

### Statistical analysis

Genotype and allele frequencies for polymorphisms were determined by gene counting. The fitness to the Hardy-Weinberg equilibrium was tested using the *χ*^2^ test. Associations between polymorphisms and risk of SFTS were estimated by use of logistic regression analyses. Odds ratios (ORs) and 95% confidence intervals (CIs) were used to measure the strength of association. In view of the multiple comparisons, the correction factor n (m − 1) (n loci with m alleles each) was applied to correct the significance level. This method showed that *P* values of 0.01 and below can be considered statistically significant after correction for multiple testing. The TNF-α serum concentrations were log transformed, and tested for differences between different groups by two-sample Wilcoxon rank-sum test. These analyses were performed using SPSS software (version 17.0, SPSS Inc., Chicago, IL). The pairwise LD calculation (Lewontin’s *D*´ and *r*^2^) and haplotype blocks construction were performed using the program HaploView 4.2. Haplotypes based on the polymorphisms in the *TNF-*α gene were inferred using PHASE 2.1 software. Haplotype frequencies of the cases and controls were compared using *χ*^2^ tests. The haplo.glm program was then used to calculate adjusted ORs for each haplotype, and the number of simulations for empirical *P* values was set at 1000.

## Supporting information

S1 Table Association between severe fever with thrombocytopenia syndrome and *TNF-α*G-238A polymorphism stratified by potential risk factors. (DOCX)

S2 Table Association between severe fever with thrombocytopenia syndrome related death and *TNF-α*T-1031C and G-238A polymorphisms stratified by potential risk factors. (DOCX)

S3 Table Primers used in polymerase chain reaction direct sequencing. (DOC)
