# Supplementary material for: Polymorphisms and haplotypes in the promoter of the TNF-α gene are associated with disease severity of severe fever with thrombocytopenia syndrome in Chinese Han population
Source: PLoS Negl Trop Dis. 2018 Jun 25;12(6):e0006547. doi: 10.1371/journal.pntd.0006547 (PMC6034906; doi:10.1371/journal.pntd.0006547)
Supplement: S1 Table — (DOCX) [file pntd.0006547.s001.docx]

**Supplemental Table 1.** **Association between severe fever with thrombocytopenia syndrome and** ***TNF-α*G-238A polymorphism stratified by potential risk factors**

| Category | GG^a^ | GA + AA^a^ | OR (95% CI)^b^ | *P* value^b^ | *P*_Homogeneity_^c^ |  |
| --- | --- | --- | --- | --- | --- | --- |
| Sex |  |  |  |  |  | |
| Male | 388/313 | 24/34 | 0.57 (0.31-1.04) | 0.06 | 0.91 | |
| Female | 534/259 | 30/27 | 0.55 (0.31-0.98) | 0.044 |  | |
| Age (years) |  |  |  |  |  | |
| ≤ 60 | 434/418 | 20/49 | 0.42 (0.24-0.74) | 0.002 | 0.07 | |
| > 60 | 488/153 | 34/12 | 0.89 (0.45-1.77) | 0.75 |  | |
| Underlying medical conditions |  |  |  |  |  | |
| With | 257/125 | 13/13 | 0.54 (0.23-1.30) | 0.17 | 0.95 | |
| Without | 666/446 | 41/48 | 0.56 (0.35-0.90) | 0.017 |  | |

NOTE: The number of genotyped samples varies because of genotyping failure for some individuals.

Abbreviations: OR, odds ratio; CI, confidence interval; NA, not applicable.

^a^ Number of genotype in hospitalized SFTS patients/number of genotype in asymptomatic/mild SFTSV-infected subjects.

^b^ ORs were calculated by logistic regression with the major allele homozygotes and heterozygotes at both polymorphisms as the reference group, and were adjusted for adjusted for age, sex, and underlying medical conditions where appropriate within the strata.

^c^ For difference in ORs within each stratum (i.e. *P* value for homogeneity).
