# Supplementary material for: Polymorphisms and haplotypes in the promoter of the TNF-α gene are associated with disease severity of severe fever with thrombocytopenia syndrome in Chinese Han population
Source: PLoS Negl Trop Dis. 2018 Jun 25;12(6):e0006547. doi: 10.1371/journal.pntd.0006547 (PMC6034906; doi:10.1371/journal.pntd.0006547)
Supplement: S3 Table — (DOC) [file pntd.0006547.s003.doc]

**Supplemental Table 3. Primers used in polymerase chain reaction direct sequencing**

| Primers | Sequences | Usage |
| --- | --- | --- |
| F1 | 5´-CTCAAAgggAgCAAgAgCTg-3´ | PCR |
| R1 | 5´-TAgCTggTCCTCTgCTgTCC-3´ | PCR |
| F2 | 5´-gTCTCCgggTCAgAATgAAA-3´ | Sequencing |
| R2 | 5´-gACCCggAgACTCATAATgC-3´ | Sequencing |

Abbreviations: PCR, polymerase chain reaction.
